# Ethanol extract from *Moringa oleifera* leaves modulates brown adipose tissue and bone morphogenetic protein 7 in high-fat diet mice

**DOI:** 10.14202/vetworld.2021.1234-1240

**Published:** 2021-05-21

**Authors:** Mas Rizky A. A. Syamsunarno, Fenty Alia, Neni Anggraeni, Vanessa Ayu Sumirat, Suhendra Praptama, Nur Atik

**Affiliations:** 1 Department of Biomedical Sciences, Faculty of Medicine, Universitas Padjadjaran, 45363, Indonesia; 2 Central Laboratory, Universitas Padjadjaran, 45363, Indonesia; 3 Working Group of Medical Genetics, Faculty of Medicine, Universitas Padjadjaran, 45363, Indonesia; 4 Study Program of Biomedical Engineering, School of Electrical Engineering, Telkom University, 40257, Indonesia; 5 Medical Laboratory Technologist, Bakti Asih School of Analyst, Bandung, 40192, Indonesia; 6 Study Program of Magister of Biotechnology, Postgraduate School, Universitas Padjadjaran, 40132, Indonesia

**Keywords:** bone morphogenetic protein 7, brown adipose tissue, high-fat diet, *Moringa oleifera* leaves extract

## Abstract

**Background and Aim:**

Brown adipose tissue’s (BAT) ability to increase energy expenditure has become a new focus in obesity research. The amount and activity of BAT are inversely correlated with body-mass index and body fat percentage. Bone morphogenetic protein 7 (BMP7) plays a role in the differentiation and development of BAT, which can be increased by bioactive compounds from several medicinal plants. *Moringa oleifera* (MO) leaves are rich with vitamin, minerals, and bioactive compounds and have been used for treating obesity-related diseases in the past. The aim of this study was to explore the potency of MO leaf extract (MOLE) to modulate BAT differentiation in mice fed a high-fat diet (HFD).

**Materials and Methods:**

Twenty-four, 5-week-old male Deutsche Denken Yoken mice (*Mus musculus*) were randomly divided into four groups: The normal chow diet group was fed a normal diet, the HFD group was fed a HFD, the HFD+MOLE1, and the HFD+MOLE2 groups were fed HFD and MOLE in a dose of 280 and 560 mg/kg body weight (BW)/day, respectively. The experiment was performed for 7 weeks. At the end of the experiment, histological analysis was performed on the interscapular BAT, and blood was drawn for BMP7 protein levels.

**Results:**

After 7 weeks, BAT weight in the HFD group was nearly twice in the weight of the HFD+MOLE1 group (125±13.78 mg vs. 75±13.78 mg; p<0.001). There was also a significant increase in BAT cell density in the HFD+MOLE1 group. BMP7 serum protein levels were significantly higher in the HFD+MOLE1 group compared to the HFD group.

**Conclusions:**

The administration of MOLE in a dose of 280 mg/kg BW/day in HFD-mice induces BAT differentiation and proliferation by upregulating BMP7 protein levels.

## Introduction

Obesity is a risk factor for various diseases, including cardiovascular diseases, hypertension, and metabolic diseases [1]. Obesity is caused by high caloric intake and sedentary lifestyles [2]. There are multiple weight loss approaches, including diet, physical activity, pharmacological therapy, and bariatric surgery for extreme obesity. A recent study suggested that stimulation of adaptive thermogenesis tissue could effectively reduce body weight (BW) [3]. In adaptive thermogenesis, the mitochondria in skeletal muscles and brown adipose tissue (BAT) produce heat as a protection against cold exposure or to regulate the energy balance from overfeeding [3]. BAT functions primarily as energy disposal units, whereas white adipose tissue (WAT) acts as energy storage units. BAT is multilocular lipid droplets with dense mitochondria, and uses lipid and glucose as fuel to produce heat via uncoupling protein 1 (UCP1) located in the inner membrane of the mitochondria [4,5]. UCP1 is the essential protein in non-shivering thermogenesis because it “uncouples” oxidative phosphorylation from adenosine triphosphate (ATP) production to release heat rather than ATP [6]. Humans and rodents have two types of BAT: The classical or constitutive BAT, and the recruitable or beige adipocytes. Classical BAT has a homogenous, multilocular morphology, and is derived from mesenchymal stem cells that expressed myogenic factor 5.

Bone morphogenetic protein 7 (BMP7) is a member of the transforming growth factor-β (TGF-β) superfamily and is expressed in various organs, including adipose tissue [7,8]. In adipose tissue, BMP7 is primarily produced by stromal vascular cells and expressed early in brown adipogenesis [9]. BMP7 plays a role in the differentiation and development of BAT by inducing the early regulators of BAT adipogenesis, *Protein domain containing 16* (PRDM16), and *peroxisome proliferator-activated receptor coactivator 1-α* (PGC-1*α*) [10]. BMP7 promotes BAT thermogenic activity by increasing UCP-1 expression and the transcription factors PPAR*γ*, CCAAT/enhancer-binding protein (C/EBPs), and *fatty acid-binding protein 4* (FABP4) [10]. The ability of BMP7 to increase BAT mass and to drive the differentiation of BAT precursors residing in skeletal muscles and WAT may be a new approach for obesity therapy [11]. In one study, serum BMP7 and insulin secretion were found to have positive correlations in non-diabetic populations [12]. This suggests that BMP7 stimulates insulin secretion and improves pancreas function [12]. In HFD-fed mice, the administration of 0.72 mg recombinant BMP7 per day for 4 weeks improved lipid-overload by inducing the fatty acid transporters CD36 and CPT1, which further increased fatty acid uptake and oxidation in BAT [13]. BMP7 also plays a role in appetite regulation. In *ob/ob* mice, the administration of BMP7-expressing adenovirus increased energy expenditure and decreased food intake, subsequently reducing BW by activating the central mTOR pathway in the hypothalamus, thus suggesting a leptin-independent pathway [14].

Phytochemical compounds, especially the polyphenols and flavonoids found in fruits and vegetables, have the potential to activate BAT. UCP1 can be regulated by flavonoids through the AMPK-activated sirtuin 1 (SIRT-1)-PGC-1*α* pathway, and by regulating PRDM16 with PPAR, C/EBP*α* and β, PGC-1, and SIRT1 during differentiation [15]. For example, 3,3′,4′,5,7-pentamethylquercetin (PMQ), the natural flavonoid found in sea buckthorns (*Hippophae rhamnoides*), can protect mice against high-fat-diet-induced obesity and increase BAT weight. In addition, BAT-selective genes may be upregulated in epididymal fat tissues, such as *Ucp1* and *Bmp7* [16]. A compound contained in green tea extract, epigallocatechin gallate (EGCG), may decrease fat storage in BAT and WAT, increase BAT density, induce the browning process in subcutaneous WAT, and reduce the whitening process in BAT when administered to high-fat diet (HFD)-induced obese mice for 8 weeks [17]. The consumption of catechin-rich beverages for 12 weeks also increased BAT density and decreased extramyocellular fat in humans [18].

*Moringa oleifera* (MO) is a fast growing, soft wood tree found in tropical and subtropical regions, including Asia, Africa, and the Middle East [19]. MO leaves are rich in vitamins, minerals, and bioactive compounds, including flavonoids, alkaloids, glucosinolates, isothiocyanates, condensed tannins, hydrolysable tannins, and saponins [19-21]. Myricetin, kaempferol, and quercetin are the main flavonoids found in MO leaves [19,22]. Dried MO leaves contain 2.090-12.200 mgGAE/100 g total polyphenol and 5.059-12.16 mg/g total flavonoids [19]. Maceration of the dried leaves with 70% ethanol results in 12.23 total phenolic and 6.20 g/100 g total flavonoids [23]. The main flavonoids in dried MO leaves were 0.04 mg/g kaempferol, 5.804 mg/g myricetin, and 0.281 mg/g quercetin [22]. The higher amounts of phenolic acids (gallic, chlorogenic, ellagic, and ferulic acid) and flavonoids (kaempferol, quercetin, and rutin) in MO leaves scavenged free radicals and protected against oxidative stress by decreasing lipid peroxide, increasing glutathione levels, and normalizing the antioxidant enzyme levels of superoxide dismutase (SOD) and catalase (CAT) in CCl4 intoxicated rats [24]. MO leaf extract (MOLE) has been used to treat obesity-related diseases (e.g., hypercholesterolemia, hypertension, hyperglycemia, type 2 diabetes, insulin resistance, non-alcoholic fatty liver disease, cancer, and inflammation) [20]. Obese rats treated with MO leaves powder for 30 days reduced serum cholesterol, triacylglycerides, very low-density lipoprotein, low-density lipoprotein, and the atherogenic index with increased high-density lipoprotein levels [25]. Treatment with MOLE for 49 days significantly reduced BW, total cholesterol, triglyceride, LDL, liver biomarkers, and blood glucose levels in rats fed HFD [26]. The rectal temperature of MOLE-treated-rats was also increased, suggesting that MOLE caused thermogenic effects [26]. In human adipose-derived stem cells, the administration of MO extract during adipogenic differentiation induced thermogenic effects through the overexpression of SIRT-1, PPAR*α*, PGC-1*α*, and UCP1 [27].

This study aimed to explore the role of MOLE in modulating BAT differentiation by upregulating BMP7 protein levels.

## Materials and Methods

### Ethical approval

The animal experimental protocol was approved by The Research Ethic Committee Universitas Padjadjaran Bandung (Approval No. 107/UN6.KEP/EC/2019).

### Study period and location

The study was conducted from January to April 2019 at the Animal laboratory of the Faculty of Medicine and Central Laboratory of Universitas Padjadjaran.

### Plant extract and animal diet preparations

One whole MO tree, about 30 centimeters long was obtained from the PT. Moringa Organik Indonesia (Blora Regency, Indonesia). The plant was identified by Dr. Iriawati, Vice Dean of Resources from the School of Life Sciences and Technology, Bandung Institute of Technology (No. 1195/11.CO2.2/PL/2019). MO leaf powder was purchased from PT. Moringa Organik Indonesia. The powder was extracted using the maceration method with 96% ethanol (Merck, USA) and water in 1:3 ratio for 24 h, and then filtered. This process was repeated 3 times. The entire filtrate was homogenized and evaporated in a rotatory evaporator until it was one-third of the initial volume. The filtrate was frozen at −20°C for 24 h, then was dried further using the freeze-drying method until it turned into a greenish concentrate. The extract was kept in the refrigerator until treatment use.

Normal chow diet (NCD) and HFD were purchased from Animal Laboratory, Faculty of Pharmacy, Bandung Institute of Technology, Indonesia. NCD contained 40.7% protein, 29.2% fat, and 30.1% carbohydrate. HFD contained 38.7% protein, 56.3% fat, and 5% carbohydrate.

### Animal model and sample collection

Twenty-four male, 5-week-old white mice (*Mus musculus*; strain Deutsche Denken Yoken) with BW between 20 and 25 g (22.0±1.5 g) were purchased from PT. Biofarma (Bandung, Indonesia). Male mice were used in this study to avoid result bias due to sex-based differences in genes and hormones [28]. The mice were housed at room temperature with a 12/12 h light/dark cycle in the Animal Laboratory at the Faculty of Medicine, Universitas Padjadjaran, Bandung, Indonesia. The mice were acclimated for 2 weeks and had unrestricted access to the NCD and water before the experiment.

### Experimental design

After the acclimatization period, the mice were randomized based on BW and randomly divided into four groups. The NCD group was fed the NCD and left untreated (control). The HFD group was fed the HFD and left untreated (negative control). The HFD+MOLE1 and HFD+MOLE2 groups were fed the HFD and received an oral dose of MOLE extract (280 mg/kg BW/day and 560 mg/kg BW/day, respectively). The two doses were selected from Bais *et al*. [26], since the rats dosed with MOLE in that study had increased rectal temperatures to suggest thermogenic effects. Modifications were made according to the dose calculation conversion table from Laurence and Bacharach due to the use of a different species. The treatments were conducted for 7 weeks.

At the end of treatment, the mice were fasted overnight (over 12 h), then sacrificed. The blood samples were collected through cardiac puncture and drawn into tubes, then were allowed to stand at room temperature for more than 15 min. The clear serum samples were separated by centrifugation at 3500 rpm for 15 min. The serum samples were stored at −80°C until further use. Interscapular BAT and inguinal WAT were rapidly excised and weighed. BAT was preserved for histological examination

### Histological analysis

BAT was placed directly into 4% paraformaldehyde, then was dehydrated and embedded in paraffin. Hematoxylin and eosin staining was performed using standard protocols. The preparation slides were read under a microscope at 400 times magnification. For each slide, the microscopic images of BAT were captured in six different fields of views of the total visible area using Optilab Advance Plus microscope camera. The camera was connected to Optilab Viewer 3 Software. Both the camera and the software are manufactured by PT Miconos, Indonesia. The qualitative histological assessment of the images was done by two examiners independently to avoid bias.

Histological scores were designed to represent the density of the multilocular lipid droplets, which reflect the size of brown adipocytes. Cell size is inversely proportional to cell density. A scale from 1 to 4 was used, with 1 having the lowest density, and 4 having the highest density.

### ELISA method

BMP7 serum levels were measured using the ELISA method. Mouse BMP7 ELISA kits were purchased from FineTest^®^ (Wuhan Fine Biotech Co., Ltd, China). The experiment was performed according to the manufacturer’s instructions.

### Statistical analysis

Statistical analysis was performed using the GraphPad Prism 9.0.0 program (GraphPad Software, Inc. San Diego USA). All of the data are expressed as mean±standard deviation. One-way ANOVA was performed followed by Bonferroni’s *post hoc* to evaluate differences between groups. p<0.05 was considered statistically significant.

## Results and Discussion

BAT is a unique thermogenic organ that has become a promising target to fight obesity. The amount of BAT in the body is inversely correlated with body mass index and body fat percentages [29,30]. BAT activity is reduced in overweight or obese individuals [29,31]. Utilizing natural compounds (e.g., ephedrine from *Ephedra* spp.) that promote BAT differentiation and activity is a promising therapy to overcome obesity [3]. Polyphenol and flavonoid derivatives, such as quercetin, rutin, myricetin, EGCG, catechin, and resveratrol, are potential compounds to promote BAT development and thermogenesis *in vitro* in HFD animal models or healthy humans [15,16,32-34]. In this study, we determined the effect of MOLE in BAT differentiation in HFD-fed mice.

**Figure-1:**
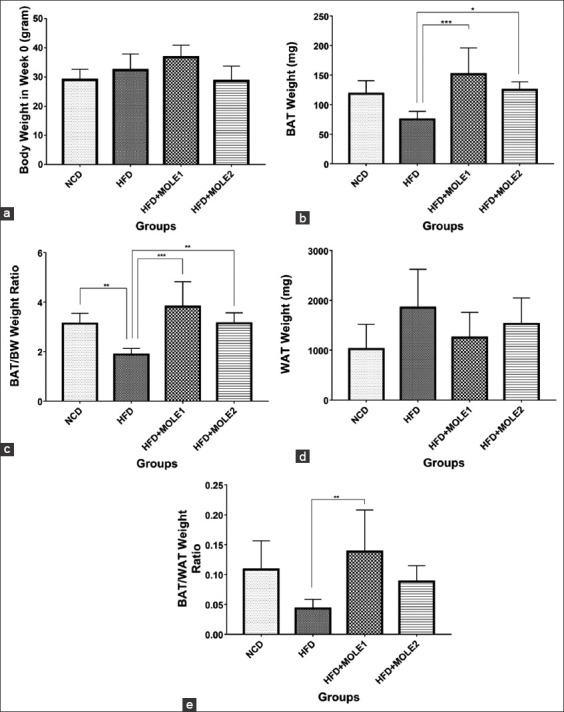
(a) Body weight (BW) after treatment (b) brown adipose tissue’s (BAT) weight, (c) BAT/BW weight ratio, (d) white adipose tissue (WAT) weight, and (e) BAT/WAT weight ratio. (*p<0.05 **p<0.01 ***p<0.001).

**Figure-2:**
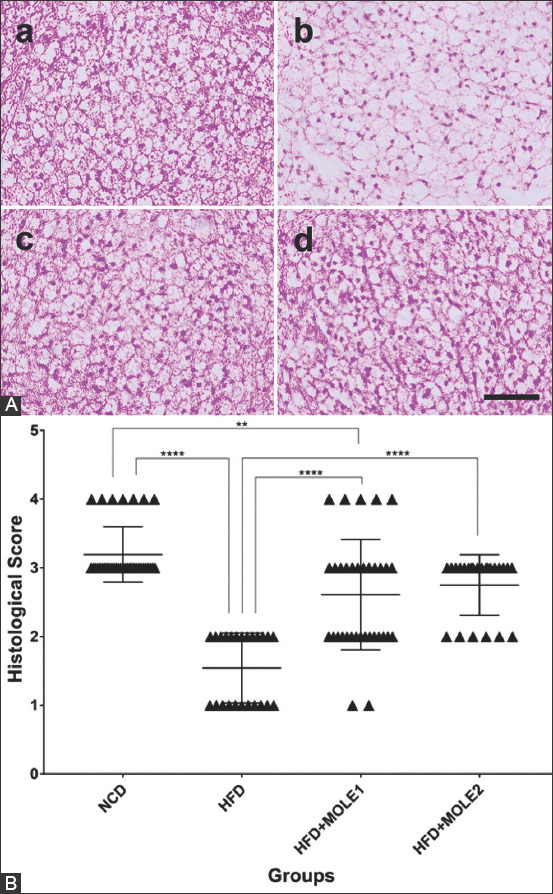
(A) Representative pictures of histopathology appearance (a=normal chow diet [NCD], b=high-fat diet [HFD], c=HFD+*Moringa oleifera* leaf extract [MOLE1], d=HFD+MOLE2) scale=50 μm and (B) histological score of brown adipose tissue’s among all groups. (**p<0.01 ****p<0.0001).

In the HFD group, BAT weights tended to decrease (Figure-1b), but there were obvious histological changes caused by the accumulation of lipids in BAT. The BAT had less dense, larger, and more ­unilocular lipid droplets (Figure-2a and b), which is similar to previous studies [17,35]. HFD reduces BAT vascularity by decreasing the expression of the vascular endothelial growth factor A (*Vegfa*) gene and proteins (a major proangiogenic cytokine), and causes capillary rarefaction and functional hypoxia in BAT [36,37]. This hypoxic state led to BAT “whitening,” an increase of mitochondria reactive oxygen species (ROS), the reduction of mitochondrial number within BAT, and the decrease of the mitochondria gene *nd5* and *Ucp1* expressions [36]. Another study showed that the BAT of HFD-fed mice was hypertrophic with increased inflammation, ER stress, ROS generation, antioxidant enzyme activities, UCP1 protein levels, and *Bmp8b* expression, but no changes were seen in the mRNA expression of the primary BAT markers *Ucp1, Zic1, Prdm16, Pgc1α, Cidea, Dio2*, *and Fgf21* [35].

The BAT weight of the HFD-mice treated with MOLE significantly increased compared to the HFD only group (HFD+MOLE1 vs. HFD+MOLE2 vs. HFD=151.7±44.46 mg vs. 125±13.78 mg vs. 75±13.78 mg; Figure-1b). This was confirmed by measuring the BAT/BW ratio and the BAT/WAT ratio (Figure-1c and 1d). The BAT of the MOLE-treated mice showed smaller lipid droplets and had a more multilocular appearance and a higher density (Figure-2a-d). The histological scoring confirmed the change in BAT density (Figure-2b). The HFD group had lower scores compared to the NCD group (1.54±0.51 vs. 3.19±0.4 [p<0.0001]). Treatment with MOLE led to higher scores compared to the HFD group (2.61±0.8 and 2.75±0.44 vs. 1.54±0.51 [p<0.0001]), suggesting an increase in the number of BAT cells. Flavonoids have the ability to stimulate BAT activity and induce the browning of WAT [38]. For example, EGCG from 0.5% green tea extract reduced the expression of the adipogenic marker adipocyte protein 2 (*Ap2*), increased the expression of the mitochondrial marker *Pgc-1α*, and the angiogenic marker vascular endothelial growth factor 165 (*Vegf165*) in the BAT of HFD-fed mice [17]. This led to improvements in vascularity and mitochondrial function, smaller lipid droplets, and higher density BAT. Another flavonoid, PMQ from sea buckthorn upregulates brown fat differentiation genes in the WAT (e.g., *Cidea, Prdm16*, and *Bmp7*) of HFD-fed mice, indicating that PMQ could also play a role in BAT differentiation. After receiving 0.04% (g/g) PMQ for 17 weeks, HFD-fed mice showed an increase in BAT weight [16].

Supplementation of 0.05% quercetin along with HFD in mice for 9 weeks upregulated UCP1 proteins and resulted in smaller brown adipocytes with smaller multiple lipid droplets in BAT compared to the BAT of the HFD-fed control group. The experiment did not provide BAT weight at the end of the quercetin supplementation, but the BW and WAT weights decreased compared to the HFD-fed control group [34], which is quite similar to the results of the MOLE-treated groups in this study (Figure-1a and d). Quercetin supplementation also promoted the browning of WAT in HFD-fed mice by upregulating the transcriptional coregulator *Prdm16* and the thermogenic factors UCP1, *Cidea*, and *Tmem26* [34]. Quercetin inhibited adipogenic differentiation by decreasing the expression of *Pparγ*, *C/ebpα*, *Fabp4*, *Ap2*, and lipoprotein lipases (*LpL***)**, and increasing the expression of BAT-like specific genes, such as *Prdm16*, *Ucp1*, *Fgf21*, T-box transcription factor 1 *(Tbx1)*, *Pgc1α*, and *Cidea* [38]. Thus, these studies enhanced the anti-obesity potential of quercetin. Other major flavonoid compounds in MOLE include myricetin and kaempferol. Myricetin induces WAT browning by activating the PGC-1*α*/irisin axis, and kaempferol increases energy expenditure and modifies the expression of metabolic genes, such as *Ucp3* and *Pgc1α*, by activating the cAMP-PKA pathway [38]. In a human study, it was found that the flavonoid catechin also increased BAT density. The daily ingestion of 540 mg catechin-rich beverage in healthy humans for 12 weeks increased supraclavicular BAT density measured using a non-invasive near-infrared time-resolved spectroscopy (NIR_TRS_) method. NIR_TRS_ was used as supportable evidence that catechin increased BAT activity/mass in humans [18].

Another proposed mechanism of how MOLE ameliorates BAT is through the suppression of inflammation in BAT. In a previous study, an increase in oxidative stress in the BAT of HFD-treated mice was measured [35]. BAT expresses Wnt10b, a canonical signaling ligand that suppresses brown adipogenesis *in vitro* and *in vivo*. The BAT of UCP1-Wnt10 transgenic mice lacked PGC-1*α* and UCP1 expressions, displayed the presence of unilocular lipid droplets, and expressed WAT genes, leading to a switch to WAT [39]. Oxidative stress activates Wnt10b signaling to further impair brown preadipocyte differentiation [39,40]. A study exploring MOLE with quercetin as its major component found that administrating 150 mg/kg BW MOLE for 15 days decreased oxidative stress and inflammation in HFD-fed mice. MOLE showed potential antioxidant activity by quenching superoxides (H_2_O_2_ and ROS), showing higher antioxidant activities (SOD, CAT, and GPx), and inhibiting the NF-kB-dependent inflammatory pathway [41]. It is expected that the ability of MOLE to reduce intracellular ROS further decreased the Wnt10b signaling. This condition would lead to BAT differentiation and adiposity, causing increased BAT density and weight in the MOLE group.

**Figure-3:**
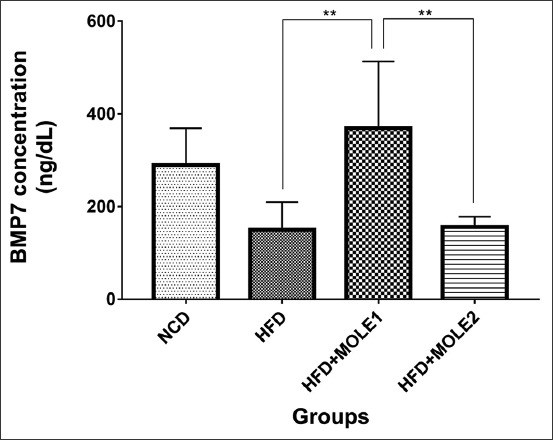
Quantification of bone morphogenetic protein 7 serum concentration with ELISA method. (**p<0.01).

BMP7 is a homodimeric protein that plays a role in the induction, development, and regulation of adipocytes, particularly BAT. The stromal vascular cells of adipose tissue synthesize BMP7 as a signaling protein [7]. BMPs and TGFβ are part of the TGF-β superfamily, which play a role in the regulation of adipose tissue differentiation, including BAT. HFD led to an imbalance of TGF-β and BMPs. TGFβ-like ligands (TGFβ, activin A, and myostatin) inhibit brown adipocyte differentiation, whereas BMP7 is a brown adipogenesis inducer. Higher TGFβ-like signals and lower BMP7 signals in obesity may inhibit brown adipocyte differentiation [31]. The administration of two different doses (200 and 400 mg/kg BW) of MOLE for 7 weeks reduced BW, improved liver biomarkers, and decreased blood glucose in HFD-fed rats [26]. Obese rats that received 600 mg/kg BW of MOLE for 12 weeks had decreased BW, downregulated mRNA expressions of leptin and resistin, and upregulated adiponectin gene expression in the visceral adipose tissue [42]. In this study, BMP7 protein levels were analyzed to determine the ability of MOLE to modulate the differentiation of BAT (Figure-3). Although there were slight decreases in the BW and the WAT weights of the MOLE groups compared to the HFD group (Figure-1a and d), a significant increase in the BAT weights in the MOLE groups indicated the improvement in the imbalance of adipose tissue differentiation regulation factors. This led to the lower TGFβ-like signaling and higher BMP7 signaling. The BMP7 serum levels significantly increased in the HFD+MOLE1 group, and slightly increased in the HFD+MOLE2 group compared to the HFD group (369.2±144.3 ng/dL and 156±22.15 ng/dL vs. 149.9±59.68 ng/dL; Figure-3). Further increases in BMP7 levels would lead to BAT differentiation and proliferation.

Based on our findings, MOLE likely modulates BAT differentiation by upregulating BMP7 proteins in the HFD mouse model. Further studies are necessary to explore the mechanism of MO to modulate BAT differentiation. The previous study suggests that PMQ, a natural flavonoid in sea buckthorn (*H. rhamnoides)*, modifies Bmp7 expression in 3T3-L1 adipocytes and epididymal adipose tissue [34]. Thus, it is also important to identify the phytochemical compounds contained in MOLE that play a role in BAT differentiation, particularly through the BMP7 downregulation pathway.

## Conclusion

This study found that the administration of MOLE in a dose of 280 mg/kg BW/day in HFD-mice induced BAT differentiation and proliferation by upregulating BMP7 protein levels.

## Authors’ Contributions

MRAAS, FA, and NT: Designed the study and wrote the manuscript. MRAAS, FA, NA, and VAS: Performed the *in vivo* experiment and collected the samples. FA, VAS, SP, and NT: Performed the histological investigation and analysis. MRAAS, FA, NA, and SP: Processed and analyzed the data. All authors read and approved the final manuscript.
